# Supplementary material for: Mediterranean swordfish (Xiphias gladius Linnaeus, 1758) population structure revealed by microsatellite DNA: genetic diversity masked by population mixing in shared areas
Source: PeerJ. 2020 Jul 28;8:e9518. doi: 10.7717/peerj.9518 (PMC7394060; doi:10.7717/peerj.9518)
Supplement: Supplemental Information 2 — N number of genotyped individuals, N A number of alleles, AR allelic richness, H o observed and H e expected heterozygotes, F IS deviations from Hardy-Weinberg equilibrium (bold identified significand deviation after Bonferroni correction). [file peerj-08-9518-s002.docx]

| Sampling Location | | Locus |  |  |  |  |  |  |  |  |  |  |  |  |  |  |  |  |  |  |  |
| --- | --- | --- | --- | --- | --- | --- | --- | --- | --- | --- | --- | --- | --- | --- | --- | --- | --- | --- | --- | --- | --- |
|  |  | (total number of alleles) | | | | | | | | | |  |  |  |  |  |  |  |  |  |  |
|  |  | Xgl,  35 | Xgl,  121 | Xgl,  561 | Xgl,  94 | Xgl,  106 | Xgl,  65b | Xgl,  74 | Xg,  Sau98R1 | Xgl,  523b | Xgl,  14 | Xg,  148b | D2A | D2B | C8 | Xg,  394 | Xg,  402 | Xg,  56 | Xg, 66 | Xg,  144 | Xg,  166 |
|  |  | (14) | (8) | (9) | (6) | (18) | (12) | (7) | (14) | (3) | (13) | (4) | (4) | (7) | (21) | (3) | (2) | (13) | (7) | (7) | (8) |
| ADR | N | 62 | 62 | 62 | 61 | 62 | 62 | 60 | 62 | 61 | 60 | 60 | 62 | 62 | 62 | 62 | 62 | 62 | 62 | 60 | 62 |
|  | N_A_ | 5 | 4 | 6 | 5 | 5 | 7 | 6 | 11 | 2 | 6 | 3 | 3 | 4 | 14 | 3 | 2 | 9 | 6 | 4 | 7 |
|  | AR | 3,2 | 3,3 | 4,8 | 4,9 | 3,5 | 6,7 | 4,4 | 9,0 | 2,0 | 4,8 | 3,0 | 3,0 | 3,2 | 9,6 | 2,1 | 1,4 | 7,8 | 4,1 | 3,4 | 6,3 |
|  | H_o_ | 0,45 | 0,55 | 0,45 | 0,64 | 0,56 | 0,68 | 0,33 | 0,87 | 0,23 | 0,58 | 0,39 | 0,37 | 0,66 | 0,94 | 0,10 | 0,03 | 0,82 | 0,23 | 0,68 | 0,77 |
|  | H_e_ | 0,44 | 0,53 | 0,49 | 0,76 | 0,57 | 0,78 | 0,65 | 0,87 | 0,25 | 0,69 | 0,39 | 0,42 | 0,62 | 0,89 | 0,09 | 0,03 | 0,79 | 0,57 | 0,68 | 0,79 |
|  | F_IS_ | -0,04 | -0,04 | 0,08 | 0,16 | 0,02 | 0,14 | **0,49** | 0,00 | 0,10 | 0,16 | 0,00 | 0,11 | ,0,08 | -0,06 | -0,03 | -0,01 | -0,04 | **0,60** | -0,01 | 0,02 |
| SIC | N | 60 | 61 | 61 | 61 | 60 | 60 | 60 | 61 | 61 | 60 | 61 | 61 | 61 | 60 | 61 | 61 | 56 | 60 | 61 | 58 |
|  | N_A_ | 5 | 5 | 6 | 5 | 5 | 8 | 4 | 11 | 2 | 6 | 3 | 3 | 4 | 13 | 2 | 2 | 8 | 6 | 3 | 7 |
|  | AR | 3,71 | 3,88 | 5,15 | 4,68 | 4,16 | 6,19 | 3,43 | 9,12 | 2,00 | 4,64 | 2,92 | 2,90 | 3,42 | 9,07 | 1,25 | 1,68 | 6,81 | 4,97 | 3,00 | 5,97 |
|  | H_o_ | 0,42 | 0,54 | 0,62 | 0,43 | 0,72 | 0,77 | 0,32 | 0,85 | 0,26 | 0,28 | 0,33 | 0,44 | 0,59 | 0,82 | 0,02 | 0,07 | 0,77 | 0,23 | 0,52 | 0,66 |
|  | H_e_ | 0,45 | 0,56 | 0,59 | 0,73 | 0,67 | 0,72 | 0,61 | 0,85 | 0,36 | 0,62 | 0,34 | 0,44 | 0,61 | 0,85 | 0,02 | 0,06 | 0,81 | 0,63 | 0,66 | 0,72 |
|  | F_IS_ | 0,07 | 0,03 | -0,05 | **0,42** | -0,08 | -0,07 | **0,48** | 0,00 | 0,27 | **0,55** | 0,03 | 0,00 | 0,04 | 0,04 |  | -0,03 | 0,05 | **0,63** | 0,20 | 0,08 |
| GRE | N | 20 | 20 | 20 | 20 | 20 | 20 | 19 | 18 | 20 | 20 | 20 | 20 | 20 | 20 | 19 | 19 | 19 | 20 | 20 | 20 |
|  | N_A_ | 4 | 4 | 6 | 5 | 5 | 6 | 6 | 8 | 2 | 6 | 3 | 3 | 3 | 9 | 1 | 1 | 6 | 4 | 3 | 5 |
|  | AR | 3,74 | 3,50 | 5,47 | 4,94 | 4,74 | 5,97 | 5,33 | 7,81 | 2,00 | 5,63 | 2,93 | 2,94 | 3,00 | 8,38 | 1,00 | 1,00 | 5,97 | 3,69 | 3,00 | 4,98 |
|  | H_o_ | 0,50 | 0,50 | 0,40 | 0,75 | 0,70 | 0,65 | 0,42 | 0,83 | 0,30 | 0,55 | 0,25 | 0,20 | 0,45 | 0,85 |  |  | 0,75 | 0,20 | 0,60 | 0,55 |
|  | H_e_ | 0,48 | 0,56 | 0,50 | 0,73 | 0,68 | 0,80 | 0,63 | 0,86 | 0,33 | 0,65 | 0,23 | 0,27 | 0,65 | 0,86 |  |  | 0,74 | 0,58 | 0,67 | 0,72 |
|  | Fis | ,0,05 | 0,10 | 0,20 | ,0,03 | ,0,03 | 0,19 | 0,34 | 0,04 | 0,09 | 0,16 | ,0,08 | 0,27 | 0,31 | 0,02 |  |  | ,0,01 | **0,66** | 0,10 | 0,24 |
| SPA | N | 85 | 85 | 85 | 85 | 85 | 85 | 83 | 84 | 85 | 83 | 85 | 85 | 85 | 84 | 85 | 85 | 84 | 84 | 82 | 81 |
|  | N_A_ | 6 | 4 | 7 | 5 | 5 | 7 | 6 | 12 | 2 | 5 | 3 | 3 | 3 | 15 | 1 | 1 | 9 | 6 | 4 | 7 |
|  | AR | 3,79 | 3,66 | 5,14 | 4,69 | 3,86 | 6,29 | 4,81 | 9,15 | 2,00 | 4,21 | 2,90 | 2,98 | 2,99 | 9,48 | 1,00 | 1,00 | 7,65 | 4,66 | 3,18 | 6,17 |
|  | H_o_ | 0,46 | 0,55 | 0,67 | 0,51 | 0,49 | 0,65 | 0,36 | 0,79 | 0,31 | 0,36 | 0,28 | 0,47 | 0,51 | 0,90 |  |  | 0,76 | 0,18 | 0,67 | 0,75 |
|  | H_e_ | 0,45 | 0,55 | 0,62 | 0,74 | 0,56 | 0,76 | 0,69 | 0,87 | 0,28 | 0,59 | 0,36 | 0,49 | 0,60 | 0,87 |  |  | 0,78 | 0,63 | 0,66 | 0,77 |
|  | F_IS_ | -0,03 | -0,01 | -0,08 | **0,32** | 0,12 | 0,14 | **0,48** | 0,10 | -0,11 | **0,38** | 0,21 | 0,04 | 0,15 | -0,04 |  |  | 0,03 | **0,72** | -0,01 | 0,02 |
|  |  |  |  |  |  |  |  |  |  |  |  |  |  |  |  |  |  |  |  |  |  |
| TIR | N | 16 | 16 | 16 | 16 | 16 | 16 | 16 | 16 | 16 | 16 | 16 | 15 | 16 | 16 | 16 | 15 | 16 | 16 | 16 | 16 |
|  | N_A_ | 4 | 3 | 5 | 5 | 4 | 6 | 2 | 10 | 3 | 4 | 3 | 3 | 3 | 8 | 2 | 1 | 7 | 4 | 4 | 6 |
|  | AR | 4,00 | 2,94 | 4,93 | 4,94 | 3,94 | 5,87 | 2,00 | 9,81 | 3,00 | 3,94 | 3,00 | 3,00 | 3,00 | 7,87 | 1,94 | 1,00 | 6,93 | 3,94 | 3,94 | 5,94 |
|  | H_o_ | 0,44 | 0,56 | 0,56 | 0,50 | 0,88 | 0,44 | 0,81 | 0,81 | 0,31 | 0,38 | 0,38 | 0,60 | 0,56 | 0,94 | 0,06 |  | 0,75 | 0,13 | 0,50 | 0,75 |
|  | H_e_ | 0,51 | 0,49 | 0,51 | 0,73 | 0,66 | 0,64 | 0,51 | 0,88 | 0,37 | 0,61 | 0,33 | 0,47 | 0,59 | 0,84 | 0,06 |  | 0,76 | 0,54 | 0,69 | 0,76 |
|  | F_IS_ | 0,15 | -0,16 | -0,11 | 0,33 | -0,33 | 0,32 | -0,61 | 0,08 | 0,17 | 0,40 | -0,13 | -0,30 | 0,04 | -0,12 |  |  | 0,01 | **0,78** | 0,28 | 0,01 |
| SAR | N | 54 | 54 | 54 | 53 | 54 | 54 | 54 | 54 | 54 | 52 | 54 | 54 | 53 | 52 | 51 | 54 | 52 | 53 | 52 | 42 |
|  | N_A_ | 6 | 5 | 7 | 5 | 5 | 7 | 6 | 11 | 2 | 7 | 3 | 3 | 3 | 13 | 1 | 2 | 8 | 4 | 4 | 6 |
|  | AR | 4,18 | 4,12 | 5,27 | 4,81 | 3,90 | 6,16 | 5,04 | 9,01 | 2,00 | 4,49 | 2,92 | 2,98 | 2,99 | 9,68 | 1,00 | 1,48 | 7,36 | 3,57 | 3,29 | 5,28 |
|  | H_o_ | 0,43 | 0,50 | 0,57 | 0,60 | 0,63 | 0,61 | 0,39 | 0,89 | 0,20 | 0,31 | 0,28 | 0,37 | 0,53 | 0,81 |  | 0,04 | 0,85 | 0,15 | 0,56 | 0,38 |
|  | H_e_ | 0,41 | 0,61 | 0,58 | 0,73 | 0,63 | 0,74 | 0,70 | 0,87 | 0,29 | 0,52 | 0,32 | 0,43 | 0,59 | 0,86 |  | 0,04 | 0,78 | 0,59 | 0,64 | 0,71 |
|  | F_IS_ | -0,05 | 0,18 | 0,01 | 0,17 | 0,00 | 0,17 | **0,45** | -0,02 | 0,31 | **0,41** | 0,14 | 0,14 | 0,11 | 0,06 |  | -0,01 | ,0,09 | **0,74** | 0,14 | **0,47** |
| CAN | N | 25 | 25 | 25 | 25 | 25 | 25 | 25 | 25 | 25 | 22 | 25 | 25 | 25 | 25 | 25 | 25 | 25 | 23 | 25 | 25 |
|  | N_A_ | 13 | 6 | 8 | 6 | 15 | 11 | 7 | 12 | 2 | 12 | 4 | 4 | 7 | 17 | 1 | 1 | 13 | 5 | 6 | 7 |
|  | AR | 11,05 | 5,60 | 7,42 | 5,53 | 12,32 | 9,16 | 6,44 | 11,24 | 2,00 | 10,25 | 3,59 | 3,60 | 5,65 | 14,32 | 1,00 | 1,00 | 11,24 | 4,88 | 5,53 | 6,36 |
|  | H_o_ | 0,84 | 0,68 | 0,72 | 0,68 | 0,92 | 0,80 | 0,36 | 0,88 | 0,56 | 0,68 | 0,48 | 0,64 | 0,76 | 0,80 |  |  | 0,76 | 0,35 | 0,64 | 0,60 |
|  | H_e_ | 0,90 | 0,79 | 0,82 | 0,76 | 0,92 | 0,84 | 0,81 | 0,92 | 0,51 | 0,86 | 0,43 | 0,58 | 0,71 | 0,94 |  |  | 0,90 | 0,75 | 0,72 | 0,72 |
|  | F_IS_ | 0,06 | 0,14 | 0,12 | 0,11 | 0,00 | 0,05 | **0,56** | 0,05 | ,0,10 | 0,21 | ,0,12 | -0,11 | -0,07 | 0,15 |  |  | 0,16 | **0,54** | 0,12 | 0,17 |
